# Integrated genetic, epigenetic, and gene set enrichment analyses identify NOTCH as a potential mediator for PTSD risk after trauma: Results from two independent African cohorts

**DOI:** 10.1111/psyp.13288

**Published:** 2018-10-17

**Authors:** Daniela Conrad, Sarah Wilker, Anna Schneider, Alexander Karabatsiakis, Anett Pfeiffer, Stephan Kolassa, Virginie Freytag, Vanja Vukojevic, Christian Vogler, Annette Milnik, Andreas Papassotiropoulos, Dominique J.‐F. de Quervain, Thomas Elbert, Iris‐Tatjana Kolassa

**Affiliations:** ^1^ Clinical Psychology and Neuropsychology University of Konstanz Konstanz Germany; ^2^ Clinical & Biological Psychology, Institute of Psychology and Education Ulm University Ulm Germany; ^3^ SAP Switzerland AG Tägerwilen Switzerland; ^4^ Division of Molecular Neuroscience University of Basel Basel Switzerland; ^5^ Transfaculty Research Platform Molecular and Cognitive Neurosciences University of Basel Basel Switzerland; ^6^ Department Biozentrum, Life Sciences Training Facility University of Basel Basel Switzerland; ^7^ Psychiatric University Clinics University of Basel Basel Switzerland; ^8^ Division of Cognitive Neuroscience University of Basel Basel Switzerland

**Keywords:** candidate gene analysis, epigenetics, gene set enrichment analysis, MAGMA, NOTCH, posttraumatic stress disorder

## Abstract

The risk of developing posttraumatic stress disorder (PTSD) increases with the number of traumatic event types experienced (trauma load) in interaction with other psychobiological risk factors. The NOTCH (neurogenic locus notch homolog proteins) signaling pathway, consisting of four different trans‐membrane receptor proteins (*NOTCH1–4*), constitutes an evolutionarily well‐conserved intercellular communication pathway (involved, e.g., in cell–cell interaction, inflammatory signaling, and learning processes). Its association with fear memory consolidation makes it an interesting candidate for PTSD research. We tested for significant associations of common genetic variants of *NOTCH1–4* (investigated by microarray) and genomic methylation of saliva‐derived DNA with lifetime PTSD risk in independent cohorts from Northern Uganda (*N*_1_ = 924) and Rwanda (*N*_2_ = 371), and investigated whether NOTCH‐related gene sets were enriched for associations with lifetime PTSD risk. We found associations of lifetime PTSD risk with single nucleotide polymorphism (SNP) rs2074621 (*NOTCH3*) (*p*_uncorrected _= 0.04) in both cohorts, and with methylation of CpG site cg17519949 (*NOTCH3*) (*p*_uncorrected _= 0.05) in Rwandans. Yet, none of the (epi‐)genetic associations survived multiple testing correction. Gene set enrichment analyses revealed enrichment for associations of two NOTCH pathways with lifetime PTSD risk in Ugandans: *NOTCH binding* (*p*_corrected _= 0.003) and *NOTCH receptor processing* (*p*_corrected _= 0.01). The environmental factor trauma load was significant in all analyses (all *p* < 0.001). Our integrated methodological approach suggests NOTCH as a possible mediator of PTSD risk after trauma. The results require replication, and the precise underlying pathophysiological mechanisms should be illuminated in future studies.

## Abstract 2

Integrating genetic, epigenetic, and gene set enrichment analyses, while accounting for the environmental factor traumatic load, we identified stress‐ and memory‐associated neurogenic locus notch homolog protein (NOTCH) genes and related gene sets as potential risk mediators for the development of posttraumatic stress disorder (PTSD) after trauma. Thus, our results strengthen the presumed role of memory‐ and inflammation‐associated genes in PTSD development, and revealed a promising target for future treatment studies. Furthermore, we demonstrated the importance of traumatic load in PTSD etiology, and of an integrated approach in order to obtain a more comprehensive understanding of the functionality of PTSD‐associated markers.

## 1 INTRODUCTION

Threats to life and physical fitness, such as a serious accident, interpersonal violence, natural disaster, rape, or war (i.e., the experience of traumatic stressors), may result in mental suffering, such as posttraumatic stress disorder (PTSD) and/or depression. The stress not only affects the mind but also the body. For instance, PTSD is accompanied by an excess of inflammatory activation (for a review, see Gill, Saligan, Woods, & Page, 2009), leading to higher morbidity and mortality among individuals with PTSD and a generally lower quality of life (American Psychiatric Association, 2013; Glaesmer, Brähler, Gündel, & Riedel‐Heller, 2011; Kubzansky et al., 2014). Multiple studies demonstrated that the risk of developing a PTSD increases with the number of different traumatic event types experienced (trauma load) (Mollica, McInnes, Poole, & Tor, 1998; Neugebauer et al., 2009; Neuner et al., 2004), a concept termed building‐block effect (Schauer et al., 2003), and can reach up to 100% with extreme levels of trauma load (Kolassa et al., 2010). Different neurocognitive models on PTSD development agree on the pivotal role of a pathological trauma‐memory formation in the etiology of PTSD (Brewin, Dalgleish, & Joseph, 1996; Brewin, Gregory, Lipton, & Burgess, 2010; Ehlers & Clark, 2000; Elbert & Schauer, 2002; Foa & Kozak, 1986; Kolassa & Elbert, 2007; Rockstroh & Elbert, 2010).

Based on twin and family studies, heritability estimates for PTSD range between 30%–50% (Sartor et al., 2012; Stein, Jang, Taylor, Vernon, & Livesley, 2002; True et al., 1993). While candidate gene and genome‐wide association studies (GWAS) identified several genetic variants associated with PTSD development (for reviews, see Sheerin, Lind, Bountress, Nugent, & Amstadter, 2017; Voisey, Young, Lawford, & Morris, 2014), our understanding of the biological underpinnings of PTSD remains limited. GWAS represent an untargeted approach that tests for associations of not only one single nucleotide polymorphism (SNP) but millions of different SNPs within the genome simultaneously. However, this requires thousands to tens of thousands of individuals to provide adequate statistical power (Voisey et al., 2014). In the largest GWAS on PTSD published to date, including more than 20,000 individuals, the Psychiatric Genomics Consortium for PTSD identified shared genetic risk factors for PTSD and schizophrenia. However, none of the included gene variants reached genome‐wide significance (Duncan et al., 2018). A major shortcoming of the large‐scale meta‐analyses so far lies in the inconsistent assessment and statistical consideration of trauma load as an important environmental factor and its potential interaction with the genetic markers under investigation. In contrast to GWAS, candidate gene studies are driven by a priori hypotheses on the biological function of target genes. Testing only a limited number of markers within certain preselected genes, candidate gene studies can provide stronger statistical power than GWAS, even in smaller study populations. Accumulating evidence from these studies suggests that genetic markers that influence memory processes such as fear conditioning or episodic memory are also associated with the development of fear memories in PTSD (for a review, see Wilker, Elbert, & Kolassa, 2014).

Due to its involvement in neuropsychiatric diseases, inflammation, and memory, the gene family of neurogenic locus notch homolog proteins (NOTCH), which includes four different highly conserved receptor genes (*NOTCH1–4*), represents an interesting target for PTSD research. Besides its regulatory function of cell fate during development and adult tissue homeostasis, previous research associated the NOTCH signaling pathway with various physical (Hubmann et al., 2013; Min et al., 2014; Sibbe et al., 2012; Wieland et al., 2017) and neuropsychiatric diseases (Kong et al., 2012), possibly by regulating inflammatory processes (Quillard & Charreau, 2013; Xu et al., 2015). A growing body of research furthermore demonstrates the importance of NOTCH genes and pathways for mental diseases, for example, schizophrenia (International Schizophrenia Consortium et al., 2009), major depressive disorder, and bipolar affective disorder (Ma et al., 2015). Steine et al. (2016) recently found an association between two *NOTCH1* SNPs and the susceptibility for anxiety and depression in victims of sexual abuse. Their findings correspond well with results from in vivo and in vitro research pointing toward an impairment of fear memory consolidation by NOTCH signaling. Even though the exact mechanisms remain to be illuminated, previous findings suggest a repression of other learning‐ and memory‐regulating genes (Hallaq et al., 2015; Zhang, Yin, & Wesley, 2015) and a modulation of the effects of stress on synaptic plasticity through NOTCH (Alberi et al., 2011; Wu & Raizen, 2011). Given the involvement of NOTCH signaling in learning and memory and its association with fear reactions, it can be expected that NOTCH genes also play a role in the development of PTSD—a question that has not yet been addressed.

However, a mere candidate gene association study on NOTCH would not provide a comprehensive understanding of its role in the etiology of PTSD, since single genetic loci can only explain a small portion of the variance of disease risk (Civelek & Lusis, 2014). A pivotal reason for the small effect sizes of single genes lies in the long and complex pathway between genetic risk factors and the development of a mental disorder, which includes several intermediate biological levels. For example, epigenetic modifications, which can influence the transcriptional accessibility of the DNA without affecting the nucleotide sequence, represent an important mechanism that can alter gene expression. The most popular epigenetic pattern studied in its relation to PTSD is DNA methylation. It is by now widely accepted that epigenetic modifications represent an individual adaptation mechanism to one's environment. These changes can occur during the entire lifespan and represent a driving factor of natural aging (for a review, see Pal & Tyler, 2016). However, epigenetic modifications can also be triggered by stress, in particular following the experience of childhood maltreatment and, to a smaller extent, through traumatic experiences during adulthood (Klengel, Pape, Binder, & Mehta, 2014). Consequently, the epigenome represents an attractive target for psychophysiological investigations on NOTCH as a potential PTSD risk gene. However, as it can be assumed that, similarly to genetics, epigenetics plays only a minor role for PTSD development at extreme levels of trauma exposure, trauma load has to be considered as a covariate in epigenetic analyses.

It is also well known that polygenic diseases, such as PTSD, are caused by a complex interplay of hundreds of genes (Schadt, 2009). For a comprehensive understanding how a gene candidate mediates disease risk, it is therefore necessary to unravel the biological context in which the gene operates (Papassotiropoulos & de Quervain, 2015; Papassotiropoulos et al., 2013). Multilocus approaches, often known as pathway or gene set enrichment analyses (GSEA), could therefore be a valuable addition to candidate gene and epigenetic analyses. GSEA tests for associations of functionally related gene sets with a phenotype of interest. Therefore, genes are clustered together based on prior biological knowledge and tested against randomly drawn gene sets of the same size (Segrè, Groop, Mootha, Daly, & Altshuler, 2010; Wang, Li, & Hakonarson, 2010). Yet to the best of our knowledge, only four studies investigated the biological underpinnings of PTSD risk using pathway analytical tools. Their results point toward the involvement of genes regulating synaptic plasticity (Duncan et al., 2018), the immune system (Ashley‐Koch et al., 2015; Wingo et al., 2015), and the glucocorticoid signaling pathway (Logue et al., 2015) in PTSD development.

Using an integrated approach, the present study aimed at providing insight into whether NOTCH genes, epigenetic modifications, or associated pathways are related to an increased risk for lifetime PTSD in two independent trauma‐exposed study cohorts from East Africa.

## 2 METHOD

### 2.1 Study cohorts

This study included two independent study cohorts, namely, survivors of the war between the rebel group Lord's Resistance Army (LRA) and Ugandan governmental troops, and survivors of the Rwandan genocide in 1994. All subjects included in this study presented with nonmissing phenotypic data regarding PTSD status and trauma load, were free of signs of current alcohol or substance abuse as well as acute severe psychotic symptoms, and did not take any psychotropic medication at the time of the assessment. Furthermore, we applied stringent quality criteria for DNA extraction procedures and genetic comparability. Exclusion criteria were (a) inconsistencies between reported sex and sex inferred from genotypic data; (b) genome‐wide missing rates > 5%; (c) deviations in heterozygosity and missing rates, identified using Bayesian clustering (Bellenguez, Strange, Freeman, Donnelly, & Spencer, 2012); (d) an unusual ancestry genetic background of subjects according to the majority of the cohort, identified using Bayesian clustering (Bellenguez et al., 2012) applied on the two first principal components inferred from HapMap CEU, YRI, CHB‐JPT populations; and (e) indices for a close relationship with other individuals in the sample, as similarly described in Wilker et al. (2018). As the Ugandan sample included a large proportion of relatives, which may inflate genetic associations, we applied two different identity‐by‐descent (IBD) thresholds ($\hat{\pi }$ > 0.2, excluding one individual of each pair indicating first‐ or second‐degree relationship and $\hat{\pi }$ > 0.1, excluding one individual of up to third‐degree relatives’ pairings). Therefore, statistical analyses in the Ugandan cohort were performed on *N* = 924 (501 women, *M_age_* = 31.26, *SD_age_* = 10.74), and on *N* = 799 (439 women, *M_age_* = 31.29, *SD_age_* = 10.92) individuals, applying the more stringent IBD threshold. For the Rwandan cohort, we applied only an IBD threshold of $\hat{\pi }$ > 0.2 as the proportion of relatives was low, resulting in *N* = 371 individuals available for statistical analyses (179 women, *M_age_* = 34.65, *SD_age_ =* *5*.88). In addition, we excluded SNPs indicating a minor allele frequency (MAF) < 0.05, SNP call rate < 0.95 and deviance from Hardy‐Weinberg equilibrium (HWE) < 0.05 from the analyses. In the Ugandan cohort, *N = *644 (69.70%) of all participants fulfilled the criteria for a lifetime diagnosis of PTSD according to DSM‐IV‐TR (American Psychiatric Association, 2000) at the time of assessment, while *N* = 263 (70.89%) individuals in the Rwandan cohort met the diagnostic criteria. Furthermore, complete epigenetic and phenotypic data were available for *N* = 331 of the Rwandan individuals.

### 2.2 Materials and study procedure

The study protocols for the Ugandan cohort were approved by the Institutional Review Board of Gulu University, the Lacor Hospital Institutional Research Committee, the Ugandan National Council for Science and Technology, Uganda, and the ethics committee of the German Psychological Society (Deutsche Gesellschaft für Psychologie), while for the Rwandan cohort the University of Konstanz, Germany, and the University of Mbarara, Uganda, approved the study protocol. All participants provided written informed consent prior to study participation.

#### 2.2.1 Diagnostic interview

Demographic and clinical data were assessed during a diagnostic interview conducted by intensively trained local lay counselors (Uganda) or by lay counselors as well as international expert psychologists with the help of local interpreters (Rwanda). For the diagnosis of lifetime PTSD according to DSM‐IV‐TR (American Psychiatric Association, 2000), the Posttraumatic Stress Diagnostic Scale (PDS; Foa, Cashman, Jaycox, & Perry, 1997) was applied as an interview. The instrument was therefore translated into Luo (Northern Uganda) and Kinyarwanda (Rwanda), then back‐translated and reviewed by trained and independent interpreters to avoid any misinterpretation. Previous studies with Ugandan (Ertl et al., 2010) and Rwandan trauma survivors (Neuner et al., 2008) indicated satisfactory psychometric properties of the translated PDS versions.

The event list used for the Rwandan cohort included 36 items that covered general traumatic events and events related to armed conflicts. The event list used for the Ugandan cohort additionally included events specific to the LRA war and comprised 62 items. Both event lists were used in previous studies (e.g., Wilker, Pfeiffer, et al., 2014; Wilker et al., 2013). Participants were asked to indicate whether they were exposed to an event in the past (*yes* or *no*). The sum score of different traumatic event types experienced was calculated for each participant, as it provides a valid, reliable, and economic assessment for trauma load (Conrad et al., 2017; Wilker et al., 2015).

#### 2.2.2 Genotyping procedure

The collection of saliva samples was part of the diagnostic interview. Participants washed out their mouth with drinking water before saliva was collected using Oragene DNA self‐collection kits following the manufacturer's protocol (DNA Genotek Inc., Ottawa, ON, Canada). Samples were biologically inactivated by adding a mixture of ethyl alcohol and trometamol (DNA Genotek Inc.) and shipped to the Transfaculty Research Platform Molecular and Cognitive Neuroscience (Basel, Switzerland) under room temperature conditions. DNA extraction and individual genotyping followed standard procedures as described in the Genome‐Wide Human SNP Nsp/Sty 6.0 User Guide (Affymetrix, Santa Clara, CA). For more details on the genotyping procedure, the reader is referred to de Quervain et al. (2012).

#### 2.2.3 Epigenetic data processing

To determine methylation status in saliva‐derived buccal cells, first, DNA was extracted as described above. For a comprehensive description of the DNA preparation procedure, see Vukojevic et al. (2014). Next, DNA was treated with bisulfite using an EZ DNA Methylation‐Gold Kit (Zymo Research, Irvine, CA). The bisulfite‐converted DNA was amplified using polymerase chain reactions and hybridized to the 450 K DNA methylation array (Illumina, San Diego, CA). To quantify methylation levels, the *M*‐value method was applied, providing more valid results considering detection rate and true positive rate compared to the beta‐value method (Du et al., 2010). For more details on the 450 K DNA methylation array and data processing, see Milnik et al. (2016).

### 2.3 Statistical procedures

#### 2.3.1 Candidate gene analyses

We planned to perform a candidate gene analyses on *NOTCH1*, *NOTCH2*, *NOTCH3,* and *NOTCH4*, spanning a total of 53 SNPs detectable by the Affymetrix Human SNP‐array 6.0 according to the UCSC Human Genome Browser (Human GRCh37/hg19; Kent, Sugnet, Furey, & Roskin, 2002). However, only 26 SNPs within *NOTCH1, NOTCH2, *and *NOTCH3* passed the SNP quality criteria applied to the Ugandan cohort (i.e., MAF > 0.05, SNP call rate > 0.95, nondeviance from HWE > 0.05). None of the SNPs located on *NOTCH4* passed these quality controls. Multiple logistic regressions were conducted and tested for main effects of SNP as predictor variable and trauma load as a covariate, as well as for a SNP × Trauma Load interaction effect on lifetime PTSD risk. In line with our epigenetic analyses, we considered genotyping batch as a covariate, whereby biological samples were processed at three different assessment periods (genotyping batch) in the Ugandan cohort. Statistical significance was determined by calculating likelihood ratio (LR) tests of nested models (Harrell, 2001). Given the lack of prior biological knowledge on associations between NOTCH markers and PTSD risk, we assumed a genotypic effect for each SNP, postulating general differences between genotype groups without determination of direction. False discovery rate (FDR) was used to correct for multiple comparisons, yet for replication analyses in the Rwandan cohort, uncorrected significant results were also taken into account. We fitted the same logistic regression model as in the Ugandan cohort with the exception that genotyping batch was not included as a covariate, because the biological samples of the Rwandan cohort resulted from one single assessment period. Analyses were performed in the statistical software R version 3.4.2 (R Core Team, 2017) using the R package GenABEL version 1.8.0 (GenABEL Project Developers, 2013). For FDR correction, the R‐implemented function *p.adjust()* was used (R Core Team, 2017). To compare genotype groups with regard to demographic data, we performed Fisher's exact test for count data and a one‐way analysis of variance (ANOVA) for continuous data. In case of non‐normally distributed model residuals, the Kruskal‐Wallis *H* test was applied.

#### 2.3.2 Epigenetic analyses

Epigenetic analyses were conducted in the statistical environment R version 3.4.2 (R Core Team, 2017). Epigenetic data were available for the Rwandan cohort, comprising *N* = 331 individuals with complete epigenetic and phenotypic data. Based on the results of our genetic analyses (see Results section Analyses of NOTCH genes in the Ugandan cohort and replication in the Rwandan cohort) and in order to provide sufficient statistical power given the even smaller cohort size available for epigenetic analyses compared to genetic analyses, we restricted our epigenetic analyses to *NOTCH3* CpG sites. Furthermore, we included only CpG sites that indicated medium to large epigenetic variability in recent reliability analyses conducted by Milnik et al. (2016). Based on methylation data from Caucasians extracted from blood, the authors found enrichment of methylation quantitative trait loci (meQTLs) in CpGs with higher variation and indicated (at least in part) a genetically driven methylation at those sites. Thus, our epigenetic analyses tested for associations of lifetime PTSD risk with the methylation level of six CpG sites within *NOTCH3 *(cg16902973, cg21514227, cg09265397, cg17519949, cg08529654, cg27320207). In line with our genetic analyses, logistic regression models included trauma load as a covariate and were furthermore adjusted for age, sex, and the main sources of variation identified by principal component analysis, including batch effects. Statistical significance was determined by calculating LR tests of nested models (Harrell, 2001). In addition to uncorrected significance values, we also report FDR corrected results (R function *p.adjust()*; R Core Team, 2017). Further, we performed linear regression analyses to test whether the methylation of identified CpG sites may depend on genetic variants (meQTLs), while accounting for trauma load as a covariate.

#### 2.3.3 Genetic pathways analyses

NOTCH‐related gene sets were extracted from different online databases (Kyoto Encyclopedia of Genes and Genomes (KEGG), https://www.genome.jp/kegg/; GeneOntology (GO), https://geneontology.org/; and Reactome, https://www.reactome.org/), which were downloaded from the MSigDB (version 6.1) database (Broad Institute, https://www.broadinstitute.org/gsea/msigdb) in November 2017. Genetic pathway analyses included 19 NOTCH‐associated gene sets, of which six were obtained from the GO database, one from KEGG, and 12 from Reactome. The computations were conducted with MAGMA on raw genotype data rather than summary statistics from previously calculated GWAS, thus providing higher statistical power (de Leeuw, Mooij, Heskes, & Posthuma, 2015). Compared to other frequently used pathway software (e.g., INRICH, Lee, O'Dushlaine, Thomas, & Purcell, 2012; or MAGENTA, Segrè et al., 2010), MAGMA shows highest power at a significantly reduced calculation time. Furthermore, the overestimation of gene sets containing a large number of genes is reduced in MAGMA compared to other approaches and linkage disequilibrium structures are directly included into analyses as principal components, successfully preventing inflation of Type I error rates (de Leeuw, Neale, Heskes, & Posthuma, 2016). To calculate gene set enrichment analyses with MAGMA, we first annotated SNPs to genes, applying the same human genome build as for previous candidate gene analyses (Human GRCh37/hg19; Kent et al., 2002). Next, gene analyses were performed, using raw genotype data from the Ugandan cohort and the SNP annotation file generated beforehand. Furthermore, trauma load and dummy‐coded genotyping batch were included as covariates. MAGMA offers different baseline gene analysis models, which are sensitive to different genetic architectures, varying by gene. As the prior knowledge about distribution of association signals across NOTCH genes was limited, we decided to use the multimodel option. Thus, all three models implemented in MAGMA (principal components regression, SNP‐wise MEAN, and SNP‐wise Top 1) were computed and resulted in an aggregated *p* value, which was used for subsequent gene‐level analyses in the Ugandan cohort. The empirical multiple testing correction that is implemented in MAGMA and based on a permutation procedure was applied (10,000 permutations). Only significantly associated pathways were considered for replication analyses in the Rwandan cohort, following the same steps as described for the Ugandan cohort. The statistical significance threshold set for all analyses was *p* < 0.05.

## 3 RESULTS

All regression models testing for associations of genetic variants and CpG sites with lifetime PTSD included trauma load and, for genetic analyses, also genotyping batch as covariates. Both trauma load and genotyping batch were significant in all analyses (all *p* < 0.001).

### 3.1 Analyses of NOTCH genes in the Ugandan cohort and replication in the Rwandan cohort

As displayed in Table 1, 26 SNPs spanned by genes *NOTCH1–3 *were tested for associations with lifetime PTSD diagnosis, including trauma load as a covariate. Three SNPs surpassed the uncorrected significance threshold (all *p*_uncorrected_ < 0.05). Of those, two SNPs were located within *NOTCH2* (rs17024559, rs17024564) and one SNP was located in *NOTCH3* (rs2074621). All SNPs were in HWE (all *p* > 0.05; see online supporting information Table S1 for more detailed SNP information). No significant interaction SNP × trauma load was observed (all *p* > 0.10). None of the three SNPs remained significant after FDR correction for multiple comparisons (all *p* > 0.05).

**Figure 1:**
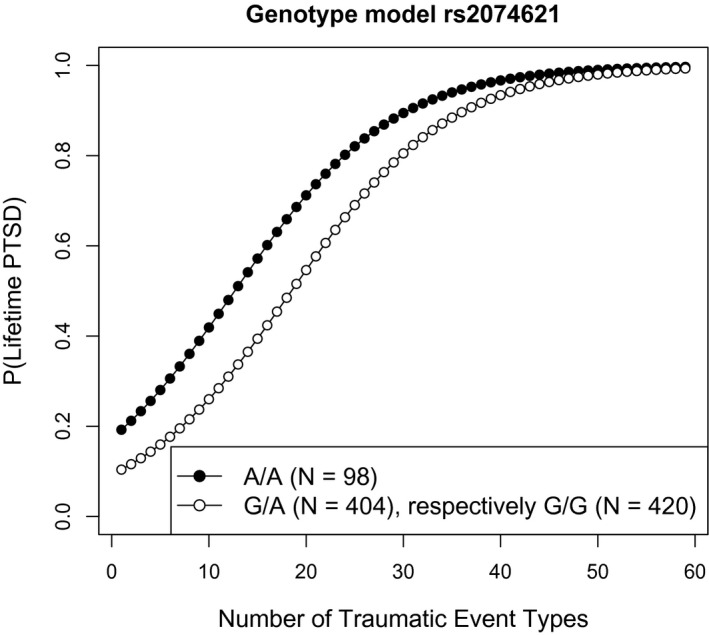
Ugandan cohort. Fitted probability values for lifetime posttraumatic stress disorder (PTSD) as a function of trauma load are plotted separately for the genotype groups of rs2074621 within *NOTCH3*. Homozygous minor allele carriers (A/A) displayed the highest risks for the development of PTSD after traumatic experiences at lower levels of trauma load, compared with G/A and G/G genotype groups. Progression curves of G/A and G/G genotype groups overlap

For replication analyses, all uncorrected significant SNPs were considered. Due to the unbalanced genotype distribution of the two SNPs located in *NOTCH2* (see Table 1), only SNP rs2074621 (*N* = 922 with complete genetic data) in *NOTCH3 *was further investigated. In the Ugandan cohort, the following genotype distribution was observed: *N* = 98 homozygote carriers of the minor A allele, *N* = 404 individuals with G/A genotype, and *N* = 420 individuals with G/G genotype. Descriptively, homozygous carriers of the minor allele (A/A) presented with higher PTSD risk at lower trauma load than heterozygotes and noncarriers, who showed a similar diminished lifetime PTSD risk in the Ugandan cohort (see Figure 1). No significant differences in demographic data existed between rs2074621 genotype groups (see supporting information Table S2). To account for the relatively large proportion of relatives in the Ugandan cohort, which may have inflated the genetic analyses results, we repeated our calculations applying a more stringent IBD threshold ($\hat{\pi }$> 0.1). Excluding one individual of each pair indicating up to third‐degree relationship, the sample comprised *N* = 797 individuals with complete genetic data for SNP rs2074621 within *NOTCH3*, which also reached uncorrected significance in this smaller cohort ($\hat{\pi }$ > 0.1: *p*_uncorrected_ = 0.03; for comparison $\hat{\pi }>$ 0.2: *p*_uncorrected_ = 0.04).

**Table 1:** Logistic regression results of *NOTCH1–3* candidate gene analyses in the Ugandan cohort

SNP	Gene	Genotype distribution			Genetics *p* value	Genetics FDR *p* value	Trauma load *p* value	Interaction Genetics × Trauma Load *p* value	Interaction Genetics × Trauma Load FDR *p* value
rs17024559	*NOTCH2*	C/C: 12	G/C: 161	G/G: 751	0.005	0.108	< 0.001	0.962	1
rs17024564	*NOTCH2*	A/A: 769	A/G: 147	G/G: 8	0.010	0.195	< 0.001	0.850	1
rs2074621	*NOTCH3*	A/A: 98	G/A: 404	G/G: 420	0.036	0.714	< 0.001	0.938	1
rs17024577	*NOTCH2*	A/A: 6	G/A: 142	G/G: 765	0.070	1	< 0.001	0.959	1
rs10127888	*NOTCH2*	C/C: 292	C/G: 452	G/G: 180	0.131	1	< 0.001	0.548	1
rs835575	*NOTCH2*	G/G: 293	G/T: 454	T/T: 177	0.134	1	< 0.001	0.622	1
rs10923931	*NOTCH2*	G/G: 294	G/T: 453	T/T: 176	0.145	1	< 0.001	0.617	1
rs2793831	*NOTCH2*	C/C: 175	T/C: 450	T/T: 283	0.172	1	< 0.001	0.551	1
rs7553305	*NOTCH2*	C/C: 39	T/C: 318	T/T: 567	0.202	1	< 0.001	0.472	1
rs3897840	*NOTCH2*	A/A: 540	A/G: 331	G/G: 53	0.207	1	< 0.001	0.910	1
rs2229971	*NOTCH1*	A/A: 95	G/A: 381	G/G: 447	0.209	1	< 0.001	0.182	1
rs10426042	*NOTCH3*	C/C: 378	C/G: 429	G/G: 113	0.255	1	< 0.001	0.855	1
rs3125009	*NOTCH1*	C/C: 240	C/T: 476	T/T: 206	0.258	1	< 0.001	0.226	1
rs2934381	*NOTCH2*	A/A: 176	G/A: 452	G/G: 290	0.264	1	< 0.001	0.600	1
rs10422818	*NOTCH3*	C/C: 789	C/T: 126	T/T: 5	0.376	1	< 0.001	0.290	1
rs3124999	*NOTCH1*	C/C: 182	T/C: 451	T/T: 283	0.528	1	< 0.001	0.754	1
rs2453044	*NOTCH2*	A/A: 199	G/A: 457	G/G: 268	0.577	1	< 0.001	0.643	1
rs7245563	*NOTCH3*	C/C: 103	T/C: 408	T/T: 386	0.608	1	< 0.001	0.775	1
rs3124596	*NOTCH1*	A/A: 469	A/G: 353	G/G: 86	0.627	1	< 0.001	0.578	1
rs10494235	*NOTCH2*	A/A: 612	A/T: 275	T/T: 31	0.669	1	< 0.001	0.670	1
rs1466708	*NOTCH2*	C/C: 28	T/C: 271	T/T: 624	0.705	1	< 0.001	0.538	1
rs10423189	*NOTCH3*	A/A: 553	A/C: 326	C/C: 42	0.806	1	< 0.001	0.745	1
rs10405248	*NOTCH3*	C/C: 111	T/C: 430	T/T: 374	0.849	1	< 0.001	0.069	1
rs7257550	*NOTCH3*	C/C: 664	C/G: 242	G/G: 18	0.893	1	< 0.001	0.469	1
rs11145770	*NOTCH1*	C/C: 70	T/C: 339	T/T: 515	0.898	1	< 0.001	0.409	1
rs3124599	*NOTCH1*	A/A: 39	G/A: 299	G/G: 582	0.952	1	< 0.001	0.610	1

Note

Results are sorted by the uncorrected *p* value for the genetic effect in decreasing order. SNP = single nucleotide polymorphism; FDR = false discovery rate.

**Figure 2:**
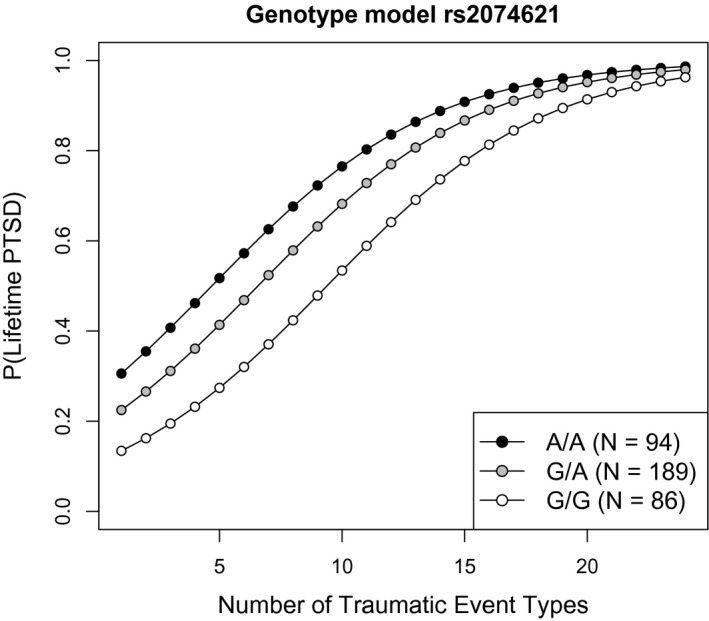
Rwandan cohort. Fitted probability values for lifetime posttraumatic stress disorder (PTSD) as a function of trauma load are plotted separately for the genotype groups of SNP rs2074621 within *NOTCH3*. As in the Ugandan cohort, homozygous minor A allele carriers displayed the highest risk for the development of PTSD after traumatic experiences. Risk was decreased in the G/A and lowest in G/G genotype group

We replicated the nominal significant association of SNP rs2074621 with lifetime PTSD risk in the Rwandan cohort (*p* = 0.02; *N* = 369 individuals with nonmissing genetic data for SNP rs2074621), where homozygous carriers of the A allele similarly displayed highest PTSD risk (Figure 2). Yet, unlike the Ugandan cohort for whom the A allele was the minor allele, the Rwandan cohort indicated the G allele as the minor allele. No differences in demographic data between the three genotype groups existed in the Rwandan cohort (Table S3).

### 3.2 Epigenetic modification of *NOTCH3 *CpG sites in the Rwandan cohort

Epigenetic analyses were based on *N* = 331 individuals with complete epigenetic data and nonmissing information on PTSD lifetime diagnosis as outcome variable. Logistic regressions were calculated for six CpG sites spanned by *NOTCH3*, previously indicated as reliably measurable (Milnik et al., 2016) and included trauma load as a covariate*. *Results showed a nominal significant association of methylation at CpG site cg17519949 with lifetime PTSD risk, *LR*(1) = 3.90, *p*_uncorrected_ = 0.05, *p*_FDR corrected_ = 0.29, yet no significant results were observed after FDR correction for multiple testing (see also Table 2). Accounting for trauma load as a covariate, we tested for SNP rs2074621 being a meQTL that potentially affects the methylation of the investigated *NOTCH3* CpG sites. We found a significant association between the methylation level at CpG site cg17519949 and the previously identified SNP rs2074621 within *NOTCH3* (SNP: b = −0.49; *F*(1, 369) = 49.66, *p* < 0.001; trauma load: *F*(1, 369) = 0.28, *p* = 0.59), whereby the level of methylation decreased with an increasing number of minor A alleles.

**Table 2:** Results of logistic regression including six reliably measurable *NOTCH3* CpG sites testing for associations with PTSD diagnostic status in the Rwandan cohort

CpG site	Infinium design type	Mapping information	Strand	UCSC CpG island name	Relation to UCSC CpG island	Statistic	*p* value	FDR *p* value
cg17519949	I	15292440	R	chr19:15292399–15292632	Island	LR(1) = 3.90	0.048	0.290
cg09265397	I	15288799	R	chr19:15288314–15288911	Island	LR(1) = 2.24	0.134	0.403
cg08529654	II	15305938	F	chr19:15306243–15307111	N_Shore	LR(1) = 1.34	0.248	0.495
cg27320207	I	15307057	R	chr19:15306243–15307111	Island	LR(1) = 0.80	0.373	0.559
cg21514227	II	15288315	R	chr19:15288314–15288911	Island	LR(1) = 0.24	0.628	0.649
cg16902973	II	15288310	R	chr19:15288314–15288911	N_Shore	LR(1) = 0.21	0.649	0.649

Note

Results are sorted by the uncorrected *p *value in decreasing order. CpG = cytosine phosphodiester guanine; Island = region with significantly increased CpG density compared to general human genome; N_Shore = region up to 2 kb upstream of a CpG island; FDR = false discovery rate.

### 3.3 Genetic analyses of NOTCH‐related pathways

Genetic pathway analyses in the Ugandan cohort were conducted with MAGMA and tested for enriched associations of 19 predefined NOTCH‐related gene sets with lifetime PTSD risk. Results indicated significant enrichment for two pathways retrieved from the GO database after correction for multiple testing (*NOTCH binding*, GO:0005112, *p* = 0.003; *NOTCH receptor processing*; GO:0007220, *p* = 0.011). Furthermore, one pathway obtained from the Reactome database showed enrichment on a trend level (*Receptor ligand binding initiates the second proteolytic cleavage of NOTCH receptor*; R‐HAS‐156988, *p* = 0.067; Table 3).

**Table 3:** Results of MAGMA gene set enrichment analysis in the Ugandan cohort

Gene set	Database	Number of contained genes	Beta	Standardized beta	Standard error	*p* value	MAGMA corrected *p* value^a^
NOTCH binding	GO	15	0.796	0.026	0.225	0.0002	0.003
NOTCH receptor processing	GO	12	0.788	0.023	0.250	0.0008	0.011
Receptor ligand binding initiates the second proteolytic cleavage of NOTCH receptor	Reactome	9	0.751	0.019	0.294	0.005	0.067
Pre‐NOTCH transcription and translation	Reactome	23	0.449	0.018	0.198	0.012	0.127
NOTCH signaling pathway	GO	78	0.229	0.017	0.105	0.015	0.153
Signaling by *NOTCH2*	Reactome	9	0.549	0.014	0.314	0.040	0.346
NOTCH HLH transcription pathway	Reactome	9	0.529	0.013	0.304	0.041	0.346
Signaling by *NOTCH3*	Reactome	9	0.500	0.013	0.315	0.056	0.435
Signaling by NOTCH	Reactome	78	0.148	0.011	0.105	0.081	0.546
NOTCH signaling pathway	KEGG	36	0.198	0.010	0.149	0.093	0.591
Signaling by *NOTCH4*	Reactome	8	0.439	0.010	0.333	0.094	0.595
Signaling by *NOTCH1*	Reactome	50	0.158	0.009	0.131	0.115	0.660
Pre‐NOTCH expression and processing	Reactome	36	0.183	0.009	0.154	0.118	0.668
Activated *NOTCH1* transmits signal to the nucleus	Reactome	19	0.237	0.009	0.208	0.126	0.693
Negative regulation of NOTCH signaling pathway	GO	15	0.242	0.008	0.249	0.166	0.780
Regulation of NOTCH signaling pathway	GO	49	0.123	0.007	0.138	0.187	0.815
*NOTCH1* intracellular domain regulates transcription	Reactome	33	0.093	0.005	0.166	0.287	0.920
Positive regulation of NOTCH signaling pathway	GO	27	0.049	0.002	0.185	0.394	0.972
Pre‐NOTCH processing in Golgi	Reactome	13	0.048	0.002	0.256	0.425	0.979

Note

Results are sorted by the corrected *p* value in decreasing order.

a Based on a MAGMA‐implemented permutation procedure (10,000 permutations).

**Figure 3:**
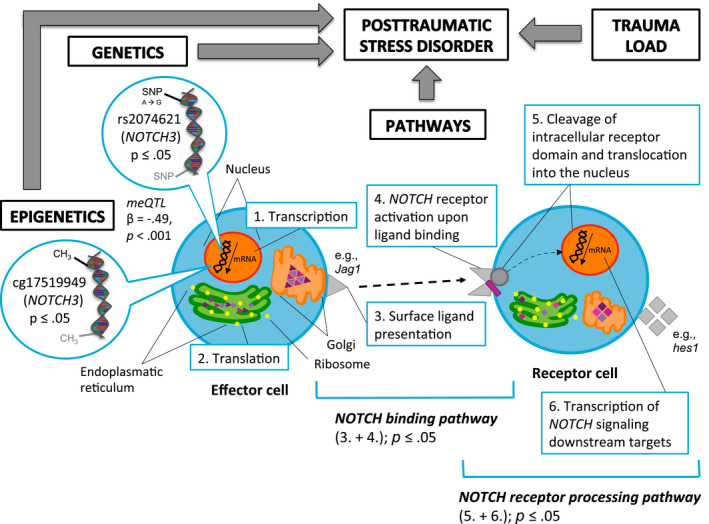
Graphic summary of the results of the integrated candidate gene association analyses, epigenetic analyses, and pathway analyses of the neurogenic locus notch homolog protein (NOTCH) family

Even though none of the above‐mentioned pathways could be replicated in the independent cohort of Rwandan genocide survivors, a positive beta for the GO pathway *NOTCH receptor processing* (GO:0007220; b = 0.22, *p*_uncorrected_ = 0.20, *p*_corrected_ = 0.31) was observed (Table S4). Figure 3 provides a graphic summary of the results of all analyses.

## 4 DISCUSSION

In line with previous studies (e.g., Kolassa et al., 2010; Mollica et al., 1998; Neugebauer et al., 2009; Neuner et al., 2004), we found a significant dose‐dependent effect of trauma load, which was included as a covariate in all analyses on PTSD risk. Moreover, this study revealed first evidence of a potential involvement of NOTCH signaling in PTSD development.

Our candidate gene analyses indicated a nominally significant association of lifetime PTSD risk with SNP rs2074621 (*N* = 922 rebel war survivors from Northern Uganda), located in an intronic region within *NOTCH3* on chromosome 19 (Human GRCh37/hg19; Kent et al., 2002). This association remained stable even after a more stringent control for the high proportion of third‐degree relatives in the cohort was applied. Furthermore, we replicated our finding in an independent cohort of *N* = 369 survivors of the Rwandan genocide. In both cohorts, homozygous carriers of the A allele descriptively presented with higher PTSD risk than G/A and G/G carriers at lower trauma load. However, differences in the minor allele (Ugandan cohort: minor A allele; Rwandan cohort: minor G allele) and unequal genotype distributions in the two cohorts led to inconsistent results for the latter two genotype groups, leaving it unclear whether the risk to develop PTSD is generally lower in G‐allele carriers or decreases with increasing numbers of “protective” G alleles. Given the involvement of NOTCH in fear memory consolidation (Dias et al., 2014), one may hypothesize that SNP rs2074621 could possibly affect the ability to store emotionally arousing memory depending on genotype, which may render homozygous A‐allele carriers more vulnerable to develop PTSD. Yet, it needs to be determined how this intronic SNP may influence memory processes and consequently PTSD risk on a biological level in detail, for example, by affecting the transcription and translation rate of downstream‐located protein coding sequences.

Corresponding to the results of the candidate gene association analyses, we identified methylation at CpG site cg17519949 (located on chr19: 15292440) within *NOTCH3* to be associated with lifetime PTSD risk on a nominal level in *N* = 331 survivors of the Rwandan genocide, controlling for the influence of trauma load. Further, we found a significant association of CpG site cg17519949 with SNP rs2074621, indicating SNP rs2074621 as a meQTL, likely to affect the methylation level of this CpG site. This assumption is further supported by the results of Milnik et al. (2016), who found enrichment of meQTLs among CpGs with medium to large epigenetic variability, as was the case with cg17519949 that is located within an exon (Human GRCh37/hg19; Kent et al., 2002) and thus could be involved in the regulation of gene expression. It is now widely accepted that NOTCH transcription and translation is negatively regulated by microRNAs, which consequently affects the intensity of NOTCH signaling (Dias et al., 2014). This is in line with Murphy et al. (2017) who showed that impaired fear extinction, as frequently observed in PTSD patients, could be rescued by targeting genes in plasticity‐associated signaling cascades (i.e., NOTCH) to increase microRNA‐controlled gene expression in the amygdala. However, their findings are based on brain tissue, and future research is needed to determine whether similar effects can be found in humans and in peripheral tissues (e.g., blood).

The results of our pathway analyses furthermore strengthened the presumed role of NOTCH in PTSD susceptibility. The significantly enriched NOTCH receptor processing pathway (GO:0007220) describes the series of successive proteolytic cleavage events following ligand binding to a NOTCH receptor, the first significantly enriched pathway (GO:0005112), at the end of which stands the expression of downstream target genes, including the hairy and enhancer of split family and related proteins. Both belong to the family of transcription repressors and thus indirectly regulate the expression of numerous NOTCH target genes. As previous research suggested that the impairment of fear memory consolidation may be driven by the repression of other learning‐ and memory‐regulating genes through NOTCH (Hallaq et al., 2015; Zhang et al., 2015), this pathway might be involved in the pathological fear memory formation in PTSD. Taken together, our GSEA suggest a potential involvement of NOTCH‐associated pathways in PTSD development and underpin the potential of pathway analytic tools for future studies on mental health conditions including PTSD, even though a high number of participants is still required to provide adequate statistical power to identify and replicate risk‐associated gene sets.

It has already been demonstrated that NOTCH is relevant in a large number of biological regulatory functions, including the immune system and the (stress‐sensitive) hematopoietic system (Oh et al., 2013). Together with its regulatory impact on fear memory consolidation (Dias et al., 2014) and long‐term memory formation (Hallaq et al., 2015; Zhang et al., 2015), mechanisms that were previously described to be altered in patients with PTSD (for reviews, see Gill et al., 2009; Wilker, Elbert, et al., 2014), one may hypothesize that NOTCH might play a role in a potential link between inflammation, pathological memory formation, and disease risk. However, the lack of previous research on NOTCH and PTSD risk in humans prevents drawing any final conclusions.

### 4.1 Strengths and limitations

This was the first study of its kind to integrate three different methodological approaches to investigate NOTCH as a potential novel mediator for PTSD risk. Yet, the exact biological mechanisms of the identified associations of NOTCH genes, epigenetic modifications, and pathways with PTSD risk remain to be illuminated by future research. Further, the generalizability of our findings and their transferability to a systematic level using different tissues (e.g., cells of the innate and adaptive immune system, neurons, and glia cells) need to be investigated.

A major limitation of this study is that not all of the presented results survived correction for multiple testing and were partially nonreplicable in an independent, smaller study cohort. Our results once more demonstrate the difficulties to detect small genetic and epigenetic effects underlying polygenic diseases like PTSD, even with targeted approaches and in cohorts with standardized assessment of traumatization and PTSD symptoms. The correction for multiple comparisons represents a justified request in genetic and epigenetic association studies to prevent Type I errors, but precludes significance of true positives on the other hand. The aim to discover minor genetic effects through exploratory testing of novel gene candidates spanning several variants leads to a dilemma between the endeavor to account for the genetic complexity of the disease and a too‐conservative control for markers to survive corrections for multiple comparisons. Even if the effect size of a risk marker is too small to reach statistical significance, it may be no less important for disease development. However, studies reporting nominally significant findings are scarce, even though some of them indicate promising associations of PTSD with neurotransmitter and neuropeptide‐related genes, among them the frequently replicated gene *SLC6A3*, which encodes the dopamine transporter (for a review, see Smoller, 2016). It therefore needs to be discussed how strict the control for multiple tests should be if the aim of the study is to identify novel PTSD risk variants that will be followed up in future studies (cf. Roback & Askins, 2005; Rothman, 1990). Instead of restricting replication to markers that pass conservative corrections for multiple testing, one might—in this case—consider the replication of nominal significant results in independent study cohorts and with multiple methodological approaches as presented in this study.

### 4.2 Conclusions and future directions

Our findings suggest an influence of NOTCH on PTSD risk in humans and strengthen the presumed role of memory‐ and inflammation‐associated genes in PTSD development. Furthermore, our study once again highlighted the importance of the environmental factor trauma load in PTSD etiology and the necessity of its consideration in genetic and epigenetic research on PTSD risk. Furthermore, we demonstrated the value of integrated genetic, epigenetic, and gene set enrichment analyses when investigating the psychophysiology of mental diseases. NOTCH has been identified to be a promising candidate to follow up in future studies on PTSD risk and treatment. For example, changes in methylation should be investigated with respect to their relevance for gene expression and protein density in the cell membrane.

## Supporting information

Table S1
Table S2
Table S3
Table S4
