# Supplementary material for: Integrated genetic, epigenetic, and gene set enrichment analyses identify NOTCH as a potential mediator for PTSD risk after trauma: Results from two independent African cohorts
Source: Psychophysiology. 2018 Oct 17;57(1):e13288. doi: 10.1111/psyp.13288 (PMC7379258; doi:10.1111/psyp.13288)
Supplement: Supplementary file 1 — Table S1 Table S2 Table S3 Table S4 [file PSYP-57-e13288-s001.docx]

**Supplement**

Supplementary Table 1.

*Detailed information on the four uncorrected significant SNPs from the initial candidate gene study, separately listed for each cohort.*

| **Cohort** | **SNP** | **Non-missing** | **Genotype distribution** | | | **Minor allele frequency**^a^ | **Hardy-Weinberg equilibrium**^b^ |
| --- | --- | --- | --- | --- | --- | --- | --- |
| **Ugandan cohort** | *NOTCH2*, rs17024559 | *N*=924 | C/C: 12 | G/C: 161 | G/G: 751 | 0.10 | *p=*.32 |
|  | *NOTCH2*, rs17024564 | *N*=924 | A/A:769 | A/G: 147 | G/G: 8 | 0.09 | *p=*.74 |
|  | *NOTCH3*, rs2074621* | *N*=922 | A/A: 98 | G/A: 404 | G/G: 420 | 0.33 | *p=*.95 |
| **Rwandan cohort** | *NOTCH2*, rs17024559 | *N*=371 | C/C: 6 | G/C: 84 | G/G: 281 | 0.13 | *p=*.92 |
|  | *NOTCH2*, rs17024564 | *N*=371 | A/A:283 | A/G: 82 | G/G: 6 | 0.13 | *p=*.98 |
|  | *NOTCH3*, rs2074621* | *N*=369 | A/A: 94 | G/A: 189 | G/G: 86 | 0.49 | *p=*.63 |

*Note:* SNP=single nucleotide polymorphism

* SNP rs2074621, which was associated with PTSD risk in the Ugandan cohort and replicated in the Rwandan cohort, is marked with an asterisk.

^a^ minor allele rs17024559=C; minor allele rs17024564=G; minor allele rs2074621=A (Ugandan cohort), respectively G (Rwandan cohort);

^b^ Chi-squares test results

Supplementary Table 2.

*Demographic overview for genotype groups of NOTCH3-SNP rs2074621 for the Ugandan cohort.*

|  | **G/G**  **(*N*=420)** | **G/A**  **(*N*=404)** | **A/A**  **(*N*=98)** | **Statistic** | ***p*-value** |
| --- | --- | --- | --- | --- | --- |
| ***N* female (%)** | 226 (53.81) | 221 (54.70) | 53 (54.08) | Fisher’s exact test^a^ | .97 |
| **Mean age (s.d.)** | 30.83 (10.94) | 31.54 (10.67) | 31.72 (10.10) | H(2)=2.17^b^ | .34 |
| **Mean trauma load (s.d.)** | 26.83 (9.41) | 26.00 (8.71) | 25.74 (7.84) | F(2,919)=1.15^c^ | .32 |

^a^ Fisher’s exact test for count data

^b^ Kruskal-Wallis H test for continuous data, as model residuals were not normally distributed

^c^ One-way analysis of variance (ANOVA) for continuous data, as model residuals were normally distributed according to Shapiro Wilk’s W test

Supplementary Table 3.

*Demographic overview for genotype groups of NOTCH3-SNP rs2074621 for the Rwandan cohort.*

|  | **G/G**  **(*N*=86)** | **G/A**  **(*N*=189)** | **A/A**  **(*N*=94)** | **Statistic** | ***p*-value** |
| --- | --- | --- | --- | --- | --- |
| ***N* female (%)** | 39 (45.35) | 89 (47.09) | 50 (53.19) | Fisher’s exact test^a^ | .51 |
| **Mean age (s.d.)** | 34.78 (7.24) | 34.56 (5.21) | 34.77 (5.90) | H(2)=0.25^b^ | .88 |
| **Mean trauma load (s.d.)** | 11.98 (4.80) | 11.95 (5.27) | 11.47 (5.34) | H(2)=0.94^b^ | .63 |

^a^ Fisher’s exact test for count data

^b^ Kruskal-Wallis H test for continuous data, as model residuals were not normally distributed

Supplementary Table 4.

Results of gene-set enrichment replication analyses in the Rwandan cohort.

| **Gene-set** | **Database** | **Number of contained genes** | **Beta** | **Standardized beta** | **Standard error** | ***p*-value** | **MAGMA corrected *p*-value^a^** |
| --- | --- | --- | --- | --- | --- | --- | --- |
| NOTCH receptor processing | GO | 12 | 0.219 | 0.006 | 0.256 | .20 | .31 |
| NOTCH binding | GO | 15 | -0.038 | -0.001 | 0.231 | .57 | .73 |

*Note:* Results are sorted by the decreasing p-value.

^a^ MAGMA-implemented empirical multiple testing correction based on a permutation procedure (10,000 permutations).
